# From Burden to Depressive Symptoms in Informal Caregivers during the COVID-19 Pandemic: A Path Analysis

**DOI:** 10.3390/ijerph18189577

**Published:** 2021-09-11

**Authors:** Tatjana Rajovic, Natasa Todorovic, Milutin Vracevic, Nina Rajovic, Andrija Pavlovic, Vedrana Pavlovic, Igor Grbic, Rosa Sapic, Slavica Krsmanovic, Marijana Vukmirovic, Tamara Stanisavljevic, Ksenija Markovic, Tanja Mostic, Dejana Stanisavljevic, Natasa Milic

**Affiliations:** 1 Institute for Medical Statistics and Informatics, Faculty of Medicine, University of Belgrade, 11000 Belgrade, Serbia; tanja.rajovic63@gmail.com (T.R.); nina.rajovic@med.bg.ac.rs (N.R.); vedrana.pavlovic@med.bg.ac.rs (V.P.); dejana.stanisavljevic@med.bg.ac.rs (D.S.); 2 Red Cross of Serbia, 11000 Belgrade, Serbia; natasa@redcross.org.rs (N.T.); milutin@redcross.org.rs (M.V.); 3 Department of Humanities, Faculty of Medicine, University of Belgrade, 11000 Belgrade, Serbia; andrija.pavlovic@med.bg.ac.rs; 4 Department for Neurology and Psychiatry, Faculty of Medicine, 38220 Kosovska Mitrovica, Serbia; gbigor86@gmail.com; 5 Department of Occupational Therapy, College for Social Work, 11000 Belgrade, Serbia; sapicdr@gmail.com; 6 Medical School, Academy of Applied Studies Belgrade, 11000 Belgrade, Serbia; slavica.krsmanovic5@gmail.com; 7 Center for Informatics and Biostatistics Belgrade, Public Health Institute, 11000 Belgrade, Serbia; jewdjiq@gmail.com; 8 Faculty of Medicine, University of Belgrade, 11000 Belgrade, Serbia; stamara8@yahoo.com (T.S.); xenia_km_96@live.com (K.M.); 9 Department of Anesthesiology, Clinical Center of Serbia, 11000 Belgrade, Serbia; tanja.mostic@gmail.com; 10 Department of Internal Medicine, Division of Nephrology and Hypertension, Mayo Clinic, Rochester, MN 55902, USA

**Keywords:** informal caregiver, the COVID-19, burden, depression, psychosocial support, mental health, path analysis

## Abstract

Background: The objective of this study was to assess the complex relationship between the multiple determinants of the caregiving process, the caregiver burden, and depression during the COVID-19 pandemic in Serbia. Methods: A cross-sectional study was conducted on a nationally representative sample (n = 798) during the COVID-19 pandemic in Serbia from March to September 2020. A nine-section questionnaire designed for this study included the characteristics of caregivers, characteristics of care and care recipients, COVID-19 related questions, and the following standardized instruments: 12-Item Short-Form Health Survey, Fatigue Severity Scale, Activities of Daily Living Scale and Instrumental Activities of Daily Living Scale, Zarit Caregiver Burden Scale, and Beck Depression Inventory. Path analysis was used for the simultaneous assessment of the direct and indirect relationships of all determinants. Results: More than two thirds (71.9%) of informal caregivers experienced a burden, and more than one quarter (27.1%) had depression symptomatology. Self-rated physical health, need for psychosocial support, and caregiver burden were the main direct predictors of depression. Multiple determinants of the caregiving process had indirect effects on depressive symptomatology via the caregiver burden as a mediating factor. Conclusions: The subjective burden presented a significant risk factor for depressive symptoms in caregivers during the COVID-19 pandemic. The provision of psychosocial support was identified as an important opportunity to reduce depressive risk in informal caregivers.

## 1. Introduction

The COVID-19 pandemic has led to an unprecedented response from health care workers, causing their mental and physical health to be stretched to the limit. The focus on front-line healthcare workers left central roles in the provision of long-term informal care in a blind spot during the crisis. Despite managing complex care situations, dealing with isolation, constant fear, and bearing a burden of responsibility to keep their families out of harm’s way, little attention and support has been received by informal caregivers [1]. In a large study assessing the consequences of the COVID-19 outbreak on informal caregivers across Europe, only 17.5% of informal caregivers felt that their work has been well-valued by society during the pandemic [2].

The effects of caregiving on caregivers’ health are individual and often extensive, and are most frequently described as distressful or as a burden [3]. The term “caregiver burden” has been introduced to describe the impact of the psychological, physical, financial, and social demands of caregiving [4]. Studies conducted before the COVID-19 pandemic identified being female, having a low educational level, living in a joint household with the care recipient, financial stress, social isolation, a longer duration of daily care, and a lack of choice in being a caregiver as risk factors for caregiver burden [5,6]. While some informal caregivers can cope with these demands, others cannot and are at greater risk of experiencing physical (health problems due to chronic stress) or psychological distress symptoms (development of depression or anxiety) [3,7,8,9,10,11,12,13,14]. Different studies have evaluated and conceptualized unique models in order to understand how caregiving stress develops and how it affects an individual [15,16,17]. 

Caregivers have been recognized as a psychologically vulnerable group [7], especially when their own needs are neglected [18]. Caregivers were more likely to develop depression than non-carers before the COVID-19 pandemic and the rate of caregivers with depression symptoms saw an increase during the pandemic (16.7% vs. 21.6%) [19]. The pandemic severely reduced and restricted access to already insufficient formal healthcare, forcing informal caregivers to undertake more responsibility under harsher conditions. Recognizing the novel set of challenges in providing informal care during the COVID-19 pandemic is of special importance for the development of comprehensive and coordinated policies and actions to support caregivers’ mental health worldwide. The objective of this study was to assess the complex relationship between the multiple determinants of the caregiving process, caregiver burden, and depression, using a path analysis, on a representative sample of informal caregivers during the COVID-19 pandemic in Serbia. 

## 2. Materials and Methods

A cross-sectional study was conducted among informal caregivers during the COVID-19 epidemic in Serbia from March to September 2020. In order to perform this study, the Faculty of Medicine of the University of Belgrade collaborated with the Red Cross of Serbia and the United Nations Population Fund. Approval was obtained from the Institutional Review Board (Ethical code: 16/2020, approved date: 23 March 2020). Participation was voluntary and anonymous. 

The study sample was selected to be nationally representative for the Republic of Serbia. A stratified cluster-sampling method was used, where each statistical region presented a stratum from which clusters of municipalities were randomly selected. For the purpose of this study, a nine-section questionnaire was designed and administrated to informal caregivers. The first parts of this questionnaire involved the sociodemographic characteristics of informal caregivers, characteristics of care, and care recipients. If care was being provided for more than one care recipient, data were collected for the most severe one. Care complexity was assessed on a scale ranging from 0 (not demanding at all) to 10 (maximally demanding). Other parts of the questionnaire included the following sections:

### 2.1. SF-12

The 12-item Short Form Health Survey [20] (Serbian version) is a self-administered questionnaire used to measure the quality of life of informal caregivers. Responses to questions are expressed on an ordinal (always to never, excellent to poor) or dichotomous (yes/no) scale. From these 12 questions, two dimensions of quality of life can be calculated: physical health and mental health (ranging from 0 to 100).

### 2.2. Fatigue Severity Scale (FSS)

The FSS [21] is a self-reported scale of nine items measuring the level of fatigue and its severity. Participants were asked to describe their degree of agreement with each statement on a seven-point Likert scale ranging from “strongly disagree” to “strongly agree”. A score of one indicated no presence of fatigue, two to four indicated moderate fatigue, and scores higher than four indicated the presence of severe fatigue. The total score was derived as mean of all these scores, with the minimum score being one and the maximum score being seven.

### 2.3. ADL and IADL

The level of independence of care recipients in performing the basic activities of daily living was measured using the Activities of Daily Living (ADL) [22] scale. The scale measures six domains of dependence in bathing, dressing, toileting, transferring, continence, and feeding. Care recipients were scored yes or no for independence in each of these six functions. The total score ranges from zero (low-dependent) to six (high-independent). 

In order to assess instrumental living skills, the Lawton Instrumental Activities of Daily Living (IADL) [22] scale was used. This scale measures eight domains of function including the ability to use the telephone, shopping, food preparation, housekeeping, laundry, mode of transportation, medication use, and handling finances. For each category, care recipients were scored for each item description that resembled their highest functional level (either zero or one). The total score ranges from zero (low-function-dependent) to eight (high-function-independent). 

### 2.4. Zarit Caregiver Burden Scale

The burden of care of informal caregivers was assessed using the 22-item Zarit Burden Interview (ZBI) questionnaire [23]. Responses to each item were scored on a five-point Likert scale, ranging from “never” to “nearly always”. Total scores of the 22 items ranged from 0 to 88. A higher score was associated with a higher informal caregiver burden and consequent distress. Scores ranging from 21 to 40 indicate mild to moderate caregiver burden, while scores from 41 to 60 demonstrate moderate to severe burden and scores from 61 to 88 indicate severe caregiver burden. Besides total burden score (22 items), two subscales were used in this study—personal strain (12 items) and role strain (6 items). The magnitude of the scale reflects two important aspects of caregiving: how stressful the experience is to one’s person (personal strain), and stress induced by role conflict or overload (role strain). 

### 2.5. Beck Depression Inventory

The Beck Depression Inventory (BDI) [24] is a 21 item self-reported scale, presented with multiple choice questions, designed to identify the presence of depression. Each inventory item refers to a specific category of depressive symptom, and the statements are ranked and weighted to represent the severity of the symptom spectrum from neutral to maximum severity. Each of the statements was assigned with numerical values from zero to three to indicate the degree of severity. Scores five to nine indicate no presence of depression, scores 10 to 18 indicate mild to moderate depression, scores 19 to 29 indicate moderate to severe depression, and scores higher than 30 indicate the presence of severe depression. The minimum total score range of the questionnaire was 0 and the highest was 63. 

### 2.6. COVID-19 Related Questions

Informal caregivers were asked to indicate how the COVID-19 outbreak impacted their health and the health of the persons they are caring for. Additional questions related to the assessment of the needs of informal caregivers during COVID-19.

### 2.7. Statistical Analysis

Numerical data are presented as a mean with standard deviation or a median with ranges. Categorical variables are summarized by absolute numbers with percentages. Path analysis was conducted to test the hypothetical effects of different characteristics of informal caregivers, characteristics of care, and care recipients on an informal caregiver’s mental health and the mediation effects of caregiver burden and providing care during the COVID-19 epidemic in Serbia. Path analysis was used as it allowed the assessment of the direct and indirect effects of the predictors through simultaneous modeling of related regression relationships. Before assessing the direct and indirect paths among the variables, the absence of multicollinearity was verified using Pearson’s correlation coefficient (r), tolerance, and variance inflation factor (VIF). The estimates were within acceptable ranges: r < 0.8, tolerance ≥ 0.1, and VIF ≤ 10. For continuous variables the skewness and kurtosis coefficients were below 1. Multiple measures were used to determine the adequacy of model-fit to the data; these included the following fit indices: the χ^2^ test, the comparative fit index (CFI), the Tucker–Lewis index (TLI), the normed fit index (NFI), and the root mean square error of approximation (RMSEA). All fit indices used to indicate the degree to which a pattern of fixed and free parameters specified in the model were consistent with the pattern of variances and covariances from a set of observed data. If the p value resulting from a χ^2^ test is greater than 0.05, the path model is considered to have a good fit. A value of the χ^2^ test less than two times its degree of freedom is considered favorable. For the RMSEA, if the value is less than 0.05, the model is considered to have a good fit. If the CFI, TLI, and NFI values are greater than 0.95, the model is considered to have a good fit. In the model, the arrows demonstrate the direction of the hypothesized association. Standardized regression coefficients are presented as path estimates, demonstrating the strength of the path between variables. To enable comparison between variables, the standardized effects were estimated to show path coefficients on a common scale ranging from −1 to 1. After controlling for other predictors in the model, the direct coefficient shows the effect of an independent variable on a dependent variable, whereas the indirect coefficient shows the effect of an independent variable on a dependent variable which is mediated by variables on the path. The sum of the direct and indirect effects is the total effect, connecting the two variables. In all analyses, the significance level was set at 0.05. Statistical analysis was done using Amos 21 (IBM SPSS Inc., Chicago, IL, USA, 2012) and IBM SPSS Statistics 25 software.

## 3. Results

A total of 798 informal caregivers from 41 municipalities in Serbia were enrolled in the study. Most of the participants were female (70.8%), and most were of age 35 to 64 (72.8%) years. Almost half of the informal caregivers were employed (47.2%), 59.4% were married, and 55.8% had had secondary education. The majority was currently taking care of one person (84.9%), and providing care alone (67.5%). The reported average physical and mental health dimension scores were 45.6 ± 11.1 and 41.0 ± 6.3, respectively. The informal caregiver’s fatigue severity scale (FSS) showed an average score of 3.5 ± 1.8, with a minimal observed value of one, and maximal value of seven. The sociodemographic characteristics, SF-12, and the FSS of informal caregivers are presented in Table 1.

Care recipients were mostly women (60.2%) older than 65 years (80.4%). More than half of care recipients had been receiving care for from 2 to 5 years (54.8%), almost every day (78.5%) and more than 12 hours a day (46.3%). Almost all of care recipients were related to the caregiver (90.6%) and 66.6% were living in a joint household. Over thirty percent (31.6%) of respondents stated having sufficient financial means, while 18.3% were receiving regular financial aid (Table 2).

More than half of caregivers (57.6%) graded care complexity as highly demanding, with observed scores ranging from seven to ten. The median ADL score of the care recipients was two (observed values range: 0–6). In performing everyday life activities, care recipients needed most assistance with bathing (61.5%), while with feeding they needed the least help (32.2%). Almost half of the care recipients needed help with dressing (48.2%), 43.5% needed assistance with transferring, and 42.1% needed help with toileting. Assistance with continence was needed in 40% of care recipients. 

The median IADL score of care recipients was seven (range of observed values: 0–8). In performing instrumental everyday life activities, phone (36%) and medication use (55.5%) were activities care recipients needed the least assistance with. Almost 90% of care recipients needed help with housekeeping and handling finances, 88.9% needed assistance with laundry, and 81.5% needed help with food preparation. Assistance with transportation and shopping was needed in 84.8% of care recipients. 

**Figure 1:**
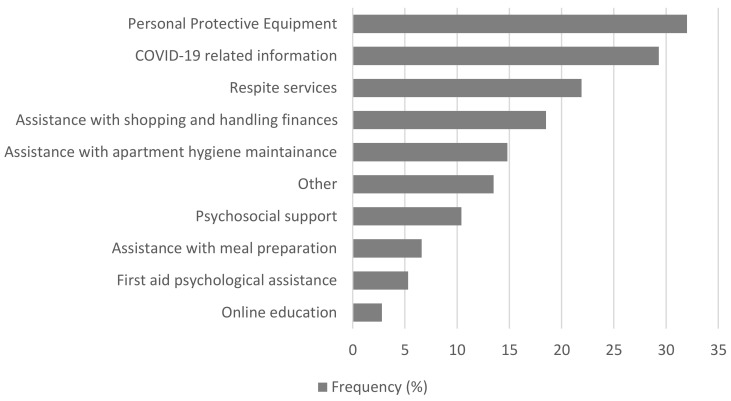
Needs of informal caregivers during the COVID-19 epidemic in Serbia.

The distribution of informal caregivers’ needs during the COVID-19 epidemic in Serbia is presented in Figure 1. The most frequent needs of informal caregivers were: personal protective equipment (32.0%), COVID-19 related information (29.3%), and respite services (21.9%) (Figure 1).

More than half (61.5%) of informal caregivers believed that their health was more at risk than before the pandemic. More than two-thirds (68.7%) believed that during the pandemic the health of the person they were caring for was more at risk than before the pandemic, and 67.3% were concerned more for their own or for the health of the person being cared for.

Based on the Zarit Caregiver Burden Scale scores, 38.7% of informal caregivers had a mild to moderate burden, 26.4% had a moderate to severe burden, and 6.0% of the respondents had a severe burden (Table 3). The mean caregiver burden of the informal caregivers was 36.6 ± 16.9 (ranging from 0 to 88), with an average personal strain value of 16.3 ± 8.6 (from 0 to 48) and a role-strain value of 8.8 ± 5.8 (from 0 to 24). Based on the Beck Depression Inventory, almost 20% of the caregivers reported symptoms of mild to moderate depression, while 7.2% experienced severe depression (Table 3).

**Figure 2:**
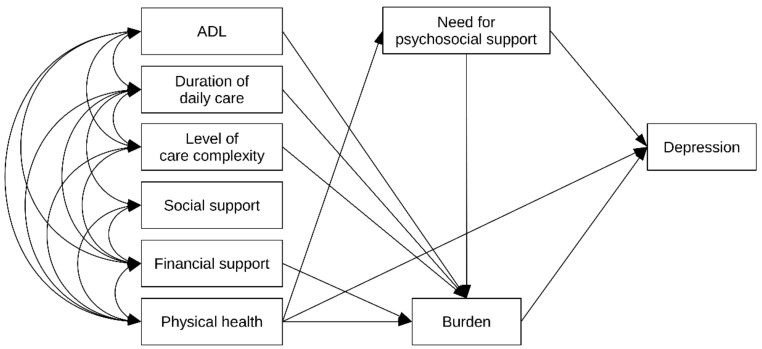
Path model presenting the complex relationship between the multiple determinants of the caregiving process, caregiver burden, and depression during the COVID-19 pandemic. ADL activities of daily living.

The hypothesized relationships among the predicting variables were tested by a path analysis, using a maximum-likelihood estimate. A standardized coefficient (B) was used to estimate the predicting effects. The best fit of the path model was achieved with χ^2^ = 3.697, df = 2, CMIN/DF = 1.849, p = 0.157, NFI = 0.997, TLI = 0.978, IFI = 0.999, CFI = 0.999, and RMSEA = 0.033. The constructed path model accounted for 35.2% of the caregiver burden, and 38.1% of depression. According to this model, self-rated physical health, caregiver burden and the need for psychosocial support during COVID 19 were the main direct predictors of a caregiver’s depression (Figure 2). Caregivers with a higher level of self-rated physical health had a lower level of depressive symptoms. Those who reported a higher level of burden and need for psychosocial support during COVID-19 had a higher level of depressive symptoms. The level of independence of care recipients in performing basic activities of daily living, duration of daily care, level of care complexity, insufficient financial support, and need for psychosocial support had important indirect effects via the burden scale (Figure 2). Beside its direct effect on depression, physical health had negative indirect effects via the burden scale and need for psychosocial support.

## 4. Discussion

The study concluded that informal caregivers had significant depressive symptoms and needed psychosocial support during the COVID-19 pandemic in Serbia. A complex relationship between the multiple determinants of the caregiving process, caregiver burden, and depression was assessed using a path analysis on a representative sample of informal caregivers during the COVID-19 pandemic in Serbia.

The majority of caregivers (70.8%) were female, 72.8% were of age 35–64 years, 86.6% had a secondary or tertiary level of education, and 84.9% were caring for one person. Similar sociodemographic distribution was presented in the Eurocarers final report [2] analyzing the impact of the COVID-19 outbreak on informal caregivers across Europe, where 80% of caregivers were women with a mean age of 57.3, highly educated (87.7%), and mostly caring for one person (77.5%). In Finland, the share of male care recipients was substantially higher (64.8%), whereas in Portugal, the majority of care recipients were female (62.4%). This corresponds to our study, where 60.2% of care recipients were female. Two-thirds were living in a joint household (66.6%) with care recipients for whom they had cared from 2 to 5 years (54.8%). The European study showed various durations of caregiving across countries, where 32.4% of respondents had provided assistance from 1 to 4 years, 27.2% from 5 to 10 years, and 35.2% for more than 10 years. Again, our study was most comparable with that from Portugal, where parents were the main category of people who received informal care (58.7% in Portugal compared to 45.8% in Serbia). Overall, in the report by Eurocarers, 30.3% of care recipients were spouses and partners, predominantly in Finland (47.7%) and Sweden (46.4%). 

Since the rise of the COVID-19 pandemic, informal caregivers have seen an increase in the intensity of care needing to be provided. The average number of weekly hours the informal caregiver spent on providing care has increased from 46.6 before the pandemic to 54.5 (+17%), with women seeing a greater increase than men [2]. In the context of having not received much support from health and social services and the many challenges posed by the COVID-19 outbreak (e.g., social isolation and containment measures), an increase in the following caregiving activities was reported: emotional support (60.3%), remote communication (49.7%), and assistance in basic and instrumental activities of daily living (13.1–44.5%) [2]. During the COVID-19 pandemic in Serbia, care recipients needed more assistance with instrumental activities than with the basic activities of daily living. Almost 90% of care recipients needed help with housekeeping, handling finances, and laundry, while 84.8% needed help with transportation and shopping. Assistance with food preparation was needed in 81.5% of care recipients. The most assistance for performing basic activities of daily living was needed for bathing (61.5%), while the least help was needed with feeding (32.2%).

In Serbia, 61.5% of informal caregivers believed that their health was more at risk than before the pandemic. In the cross-national European study, only half of the caregivers (51.5%) felt able to look after their own health and wellbeing during the pandemic [2]. Additionally, four out of five informal caregivers (78.2%) were worried about a possible decline in health of their care recipient due to the pandemic. This is supported by the results of our study, where a large number of caregivers (68.7%) believed the health of the person they were caring for was more at risk than before pandemic, and 67.3% were more concerned for their own or the health of the person being cared for. Concern about their ability to care safely due to a lack of knowledge, information, or equipment relating to COVID-19 was expressed by 41.8% of reported European caregivers [2]. The Czech Republic, Germany, and Sweden reported the lowest level of concern. Support measures, such as having free access to personal protective equipment, were recognized as important by 70.8% of the European caregivers surveyed [2]. Personal protective equipment was the most commonly reported need among caregivers in Serbia (32.0%), followed by the need for COVID-19 related information (29.3%), and respite services (21.9%). 

During the COVID-19 pandemic, more than two-thirds (71.9%) of informal caregivers in Serbia experienced a mild to severe burden, and more than one quarter (27.1%) had mild to severe depression symptomatology. Due to depression’s multifactorial nature, a complex path model was used to provide more insight into the relative importance of a caregiver’s multiple mental health predictors. Path analysis allowed the simultaneous assessment of the direct and indirect relationships of the multiple determinants of the caregiving process, caregiver burden, and depression during the COVID-19 pandemic. In the presented model, caregivers’ self-rated physical health and caregiver burden, as well as need for psychosocial support during COVID 19, were the main direct predictors of a caregiver’s depression. In addition, the significant mediating role of caregiver burden and the need for psychosocial support was also identified in the model. The level of independence of care recipients in performing basic activities of daily living (ADL), the duration of daily care, the level of care complexity, insufficient financial support, and caregivers’ self-perceived physical health had important indirect effects on depressive symptomatology via the burden scale. Results of our analysis show that caregiver burden was predicted by the physical health of the caregivers, which is in line with the results conducted by several studies, where heavier caregiver burden was proven to be associated with poorer physical health [25,26,27]. In fact, our model provided evidence that the health of caregivers is the key determinant of both burden and depression, while functioning capacity in ADL was one of the most important predictors of caregiver burden. Recent meta-analyses have identified perceived social support as a good predictor of subjective burden [28], while caregiver burden presented a significant risk factor for depressive symptoms in caregivers of older people [29]. 

Considering the impact of the pandemic on the conditions under which informal caregivers provide care, it is more than urgent to put in place adequate measures to help caregivers overcome the chronic stress they are experiencing, the feeling of subjective burden, and depression. Increasing the scope and diversity of formal services to alleviate some of the burden of care (formal home care services, respite services, training and psychological counseling) as well as providing access to preventive healthcare/mental health services are possible approaches [30]. Provision of direct psychological support to caregivers may relieve the additional stress caregivers have experienced during the COVID-19 pandemic. In a study conducted in the United Kingdom [19], a large percentage of caregivers with symptoms of depression (60%) reported having had no psychological support (online or face-to-face) during the COVID-19 pandemic, while 20% of caregivers who were diagnosed with probable depression felt they did not need psychological support. In our study, the need for psychosocial support had both direct and indirect effects (through caregiver burden) on depression symptomatology, indicating that the need for psychosocial support was recognized but not met in the new circumstances of the COVID-19 pandemic. This is rather worrying, as access to psychological support can reduce depressive risk by 43% [19]. These findings could have implications for current practice, on how the needs of this vulnerable group are properly addressed, and on ensuring that proper treatment and support, including medication and/or psychosocial support for depression, is accessible as quickly as possible [19]. The importance of focusing on wellbeing as well as reducing stress to support people’s own coping mechanisms and pliability was recognized at a conference held in Amsterdam in 2019, where 24 countries and 10 international aid organizations agreed that mental health and psychosocial support (MHPSS) must be a standard for any humanitarian response in crisis situations [31]. The Inter-Agency Standing Committee Guidelines on Mental Health and Psychosocial Support in Emergency Situations and the WHO Mental Health Gap Action Plan gave direction as to what kind of support should be made available [32]. In April 2020, members of the Latin American Regional Mental Health and Psychosocial Support Response Team presented a minimum package of recommended MHPSS interventions for COVID-19 [33]. The recommendations were organized according to the four levels of the intervention pyramid for MHPSS in emergencies: psychosocial considerations for basic services and security; community and family supports; focused, non-specialized supports; and specialized services.

Much still needs to be discussed about the effects of psychosocial interventions on informal caregivers’ mental health. A recent systematic review of the effectiveness of mental health interventions for informal caregivers of persons with dementia living at home showed positive effects on at least one of the outcomes followed in 25 of 46 studies (54.3%) [34]. Positive effects were most often (46.2%) reported for the subjective burden. In contrast, a recent Cochrane review suggested that heterogeneity across studies (n = 3725; 19 studies) makes it difficult to draw firm conclusions regarding the effectiveness of psychosocial interventions for informal caregivers of people living with cancer [35]. However, the importance of developing cooperation in creating long-term MHPSS approaches is not disputable. Sustainable, good-quality MHPSS must be a combined effort by local, national, and international communities, as well as governmental and nongovernmental institutions cooperating with caregivers and their families.

The main limitation of this study is that it was conducted early in the COVID-19 pandemic, when there was no COVID-19 vaccine available. The absence of the vaccine to protect informal caregivers and those for whom they are providing care creates psychological stress during care, thus contributing to caregivers’ depressive symptomatology. Future research should consider adding to the model variables related to COVID-19 vaccine access and the use of support services such as psychosocial support for caregivers. Trials with clear procedure evaluations along with cost-effectiveness analyses should provide more detailed MHPSS intervention descriptions.

## 5. Conclusions

Subjective burden presented a significant risk factor for depressive symptoms in caregivers during the COVID-19 pandemic and mediated the effect of the level of independence of care recipients in performing basic daily activities (ADL), the duration of daily care, the level of care complexity, insufficient financial support, and the need for psychosocial support. The provision of psychosocial support was recognized as an important opportunity to reduce depressive risk in informal caregivers.

## Figures and Tables

**Table 1:** Sociodemographic characteristics, SF-12, and fatigue severity scale (FSS) of informal caregivers.

Sociodemographic Characteristics of Informal Caregivers	n (%) *
**Female gender**	560 (70.8)
**Age**	
18–34	47 (6.0)
35–64	574 (72.8)
65+	167 (21.2)
**Marital status**	
Married	472 (59.4)
Single	101 (12.7)
Divorced	98 (12.3)
Widowed	95 (11.9)
Domestic partnership	31 (3.9)
**Level of education**	
Primary education or below	107 (13.4)
Secondary education	444 (55.8)
Tertiary education or above	245 (30.8)
**Employment status**	
Employed	376 (47.2)
Unemployed	154 (19.3)
Retired	192 (24.1)
Housewife	60 (7.5)
Other	15 (1.9)
**Caring alone**	537 (67.5)
**Number of persons cared for**	
One	667 (84.9)
More than one	119 (15.1)
**SF-12, mean ± sd, range**	
Physical health score	45.6 ± 11.1 (19–64)
Mental health score	41.0 ± 6.3 (22–57)
**Fatigue severity scale (FSS), mean ± sd, range**	3.5 ± 1.8 (1–7)

* Percentages exclude participants with missing data (<5% for all).

**Table 2:** Characteristics of care and care recipients.

Characteristics of Care and Care Recipients	n (%) *
**Age (years)**	
<18	19 (2.4)
18–65	136 (17.0)
≥65	637 (80.4)
**Female gender**	467 (60.2)
**Duration of caregiving (years)**	
≤1	188 (24.3)
2–9	437 (56.4)
≥10	149 (19.3)
**Caregiving frequency**	
Everyday	619 (78.5)
Several times a week	122 (15.5)
At least once a week	33 (4.2)
At least once a month	15 (1.9)
**Daily duration of caregiving (hours)**	
<6	295 (38.0)
6–12	122 (15.7)
>12	360 (46.3)
**Living with caregiver in joint household**	531 (66.6)
**Related to caregiver**	712 (90.6)
**Relationship with care recipient**	
Father/mother	326 (45.8)
Husband/wife	95 (13.4)
Son/daughter	61 (8.6)
Other blood relative	229 (32.2)
**Sufficient financial means**	251 (31.6)
**Regular financial aid**	145 (18.3)

* Percentages exclude participants with missing data (<5% for all).

**Table 3:** Zarit Caregiver Burden Scale and Beck Depression Inventory.

Zarit Caregiver Burden Scale	n (%)
Little or no burden	230 (28.8)
Mild to moderate burden	309 (38.7)
Moderate to severe burden	211 (26.4)
Severe burden	48 (6.0)
**Beck Depression Inventory**	
No depression	577 (72.9)
Mild depression	89 (11.2)
Moderate depression	69 (8.7)
Severe depression	57 (7.2)

## Data Availability

The data presented in this study are available on request from the corresponding author.
